# Whole-genome SNP allele frequency differences between Tibetan and Large white pigs reveal genes associated with skeletal muscle growth

**DOI:** 10.1186/s12864-024-10508-7

**Published:** 2024-06-12

**Authors:** Heli Xiong, Yan Zhang, Zhiyong Zhao, Qian Sha

**Affiliations:** Animal Nutrition and Swine Institute, Yunnan Academy of Animal Husbandry and Veterinary Sciences, Kunming, 650224 China

**Keywords:** Whole-genome SNPs, Tibetan pigs, Large white pigs, Skeletal muscle growth, Specific SNPs, Predominant SNPs

## Abstract

**Background:**

The skeletal muscle growth rate and body size of Tibetan pigs (TIB) are lower than Large white pigs (LW). However, the underlying genetic basis attributing to these differences remains uncertain. To address this knowledge gap, the present study employed whole-genome sequencing of TIB (slow growth) and LW (fast growth) individuals, and integrated with existing NCBI sequencing datasets of TIB and LW individuals, enabling the identification of a comprehensive set of genetic variations for each breed. The specific and predominant SNPs in the TIB and LW populations were detected by using a cutoff value of 0.50 for SNP allele frequency and absolute allele frequency differences (△AF) between the TIB and LW populations.

**Results:**

A total of 21,767,938 SNPs were retrieved from 44 TIB and 29 LW genomes. The analysis detected 2,893,106 (13.29%) and 813,310 (3.74%) specific and predominant SNPs in the TIB and LW populations, and annotated to 24,560 genes. Further GO analysis revealed 291 genes involved in biological processes related to striated and/or skeletal muscle differentiation, proliferation, hypertrophy, regulation of striated muscle cell differentiation and proliferation, and myoblast differentiation and fusion. These 291 genes included crucial regulators of muscle cell determination, proliferation, differentiation, and hypertrophy, such as members of the Myogenic regulatory factors (MRF) (*MYOD*, *MYF5*, *MYOG*, *MYF6*) and Myocyte enhancer factor 2 (MEF2) (*MEF2A*, *MEF2C*, *MEF2D*) families, as well as muscle growth inhibitors (*MSTN*, *ACVR1*, and *SMAD1)*; KEGG pathway analysis revealed 106 and 20 genes were found in muscle growth related positive and negative regulatory signaling pathways. Notably, genes critical for protein synthesis, such as *MTOR*, *IGF1*, *IGF1R*, *IRS1*, *INSR*, and *RPS6KA6*, were implicated in these pathways.

**Conclusion:**

This study employed an effective methodology to rigorously identify the potential genes associated with skeletal muscle development. A substantial number of SNPs and genes that potentially play roles in the divergence observed in skeletal muscle growth between the TIB and LW breeds were identified. These findings offer valuable insights into the genetic underpinnings of skeletal muscle development and present opportunities for enhancing meat production through pig breeding.

**Supplementary Information:**

The online version contains supplementary material available at 10.1186/s12864-024-10508-7.

## Introduction

Tibetan pigs (TIB) are significant contributors to pork production in high-altitude regions, because they are adaptable to high altitudes, low hypoxia and cold environments. They are primarily found on the Qinghai-Tibet Plateau, Yunnan Diqing, Sichuan Aba and Ganzi, as well as Gansu Gannan. Compared with Large white pigs (LW), TIB exhibit a greater proportion of umami amino acids and essential fatty acids, as well as a lower n6:n3 ratio [1]. These characteristics contribute to the superior meat quality, enhanced flavor, and popularity in the high-end markets [2]. However, TIB exhibit comparatively slower growth rate and smaller body size in comparison to LW [3]. According to the Chinese National Animal and Poultry Genetic Resources Pig Chronicle, TIB typically weigh approximately 25 kg at the age of 12 months, whereas LW achieve this weight at a mere two months of age. Consequently, elucidating the genetic factors underlying the disparity in skeletal muscle growth between TIB and LW would prove advantageous in enhancing the pork production of TIB in the future.

The process of skeletal muscle growth is intricate and constantly changing, encompassing various stages such as the determination of embryonic mesodermal progenitor cells to the myogenic lineage, their differentiation into myoblasts, the proliferation and fusion of myoblasts to form multinucleate myotubes, the development of myotubes filled with myofibrils to create mature myofibers, and ultimately the hypertrophy of myofibers through the addition of extra myofibrils [4, 5]. In the process of functional mature myofiber formation, multiple transcription factors are expressed in a coordinated manner. Notably, members of the Myogenic Regulatory Factors (MRFs) including MyoD, Myogenin, Myf5, MRF4, and Myocyte Enhancer Factor 2 (MEF2), specifically MEF2A, MEF2B, MEF2C and MEF2D play pivotal roles in these processes [6–9]. MyoD and Myf5 are involved in the specification of embryonic mesodermal progenitor cells into myoblasts, while myognenin (MyoG) and either MyoD or MRF4 (Myf6) are essential for the differentiation of myoblasts into myocytes. The cooperative action of MyoD and MEF2 family members is responsible for the activation of transcription of a majority of skeletal muscle genes, including *M-creatine kinase* (*CKM*), m*yosin heavy chain* (*MYH*), and *desmin* (*DEM*) [6, 7, 9]. Muscle growth occurs through hypertrophy of existing myofibers, achieved by the addition of myofibrils to increase muscle mass, and the addition of sarcomeres to the ends of existing myofibrils to increase their length during the postnatal stage [9, 10]. In both scenarios, the synthesis and deposition of new structural and contractile proteins, such as desmin, myosin, actin, troponin, and tropomyosin are necessary within the myofibers [9, 10]. The mTOR signaling pathway and the myostatin-Smad2/3 pathway are two major signaling pathways that control protein synthesis, and they act as positive and negative regulator for muscle growth, respectively [11]. The mTOR signaling pathway is crucial for regulating protein synthesis, with upstream activators including growth factors such as IGF1 and insulin, acting through the PI3K-Akt cascade, as well as various amino acids acting via Rag GTPases [12]. Myostatin, a member of the transforming growth factor (TGF)-β family, is involved in inhibiting postnatal muscle fiber hypertrophy by suppressing Akt activation, and subsequently the mTORC1 signaling pathway in a Smad2/3-dependent manner [11, 13].

Whole-genome sequences has been widely used to identify genetic basis attributing to divergent traits between breeds on a wide range of organisms, such as high and low fat deposition in sheep tail [14], small and large body size in chicken [15], dark and white pigmentation in duck [16], slow and fast muslce growth [17] and high and low adipose depostion in pig [18]. Selective sweep analysis is the commonly employed method to reveal the genetic basis of extreme breeds based on whole-genome sequences. Several methods can be employed for detecting sweeps, such as site frequency spectrum based methods (Tajima’s D; Fay and Wu’s H statistic), linkage disequilibrium based methods (extended haplotype homozygosity, EHH; integrated haplotype score, iHS), methods based on reduced local variability (runs of homozygosity, ROH), single-site population differentiation (Fixation index, *F*_ST_), and haplotype-based differentiation methods (cross-population extended haplotype homozygosity, XP-EHH; haplotype-based extension of the FLK statistic, hapFLK) [19]. Those methods use an empirical cutoff value, e.g. the top 1% or 5% of extreme high values, to determine the candidate genes. However, it’s challenging to determine which empirical value is a better or correct choice. The theory of animal genetics posits that the frequencies of alleles between specific breeds intrinsically determine the underlying cause of the phenotypic differences between them [20]. The formula *µ = α (p-q) + 2pqd* defines the relationship between the population phenotypic value and the allele frequency. In this equation, *α* represents the additive effect, *p* represents the allele frequency of the synergetic allele, *q* represents the allele frequency of the reduced allele, and *d* represents the deviation caused by the dominant effect [20]. In the absence of allele interactions, it can be inferred that when the allele frequency (*p*) of a causal variation exceeds 0.50 (*d = 0*) or 0.30 (*d = α*), the population phenotype value (*µ*) will surpass the average level.

Thus, in this study, we screened SNP allele frequency to detect specific and predomiant SNPs in the TIB (slow growth) and LW (fast growth) breeds to identify genes associated with skeletal muscle development. A total of 291 genes were found involved in the biological process of skeletal muscle development, and 106 and 20 genes were identified in positive and negative regulatory pathways for skeletal muscle growth. This sduty comprehensively detected the potential SNPs associated with muscle growth difference observed between TIB and LW populations and provided valuable insights into the genetic underpinnings of skeletal muscle development.

## Materials and methods

### Whole-genome sequence and SNP calling

Blood samples were collected from 5 Diqing TIB pigs and 15 LW pigs from Lanhua and Yongsheng Farms in Yunnan Province. Samples were obtained from the jugular vein and rapidly frozen at − 20 °C. Blood DNA was extracted using a DP304 kit (TIANGEN). DNA purity (OD260/280 ratio) was assessed using a Nanodrop2000 Spectrophotometer (Thermo Fisher Scientific); Qubit was used for precise quantification of DNA concentration; DNA samples with OD values between 1.8 and 2.0 and a concentration of over 1 µg were utilized for library construction. After meeting the library preparation requirements, the DNA was fragmented via ultrasonication. Then, the fragmented DNA was subjected to fragment purification, end repair, A-tailing, sequencing adaptor ligation, and library construction. The constructed libraries were sequenced using a HiSeq X Ten (Illumina, CA, USA) with a read length of 150 bp. Sequencing was performed using an Illumina NovaSeq™ 6000 platform in 150 bp paired-end sequencing mode. In addition, publicly shared raw sequencing data from the NCBI SRA database for 39 TIB pigs from Tibet, Gansu, Yunnan, and Sichuan provinces and 14 LW pigs were merged (Supplementary Table S1) to generate a more comprehensive dataset for comparison. The 39 TIB pigs from Tibet, Gansu, Yunnan, and Sichuan Province were confirmed to have little or no introgression from LW pigs based on population structure analysis in our previous study. The raw sequencing data were filtered to remove reads with adapters and low-quality reads (the number of bases with quality value Q < = 15 accounted for more than 40% of the entire read) and low quality bases (quality value Q < = 20). We downloaded the pig genome Sscrofa11.1 sequence from NCBI (https://www.ncbi.nlm.nih.gov/data-hub/assembly/GCF_000003025.6/) for use as a reference genome for subsequent analysis. Next, Burrows-Wheeler alignment (BWA, v0.7.5a-r405) [21] was used to align the sequencing data to the reference genome. Picard software (http://broadinstitute.github.io/picard/, v1.94) was used to remove reads derived from PCR duplicates, and uniquely aligned data were retained for subsequent genome variation detection. Finally, the HaplotypeCaller module in GATK was used for SNP detection [22]. High-quality SNPs were obtained with the following filtering parameters: QD < 2.0, MQ < 40.0, FS > 60.0, QUAL < 30.0, MQrankSum < − 12.5, ReadPosRankSum < − 8.0, -ClusterSize 2, -ClusterWindowSize 5, and biallelic SNPs with no more than a 10% missing rate. High-quality SNPs were further annotated using SNPeff (v4.3) with parameter ‘-ud 1000’ based on the gene annotation of the pig Sscrofa11.1 reference genome [23].

### Detecting TIB-specific and predominant SNPs

The relationship between the population phenotypic value and the allele frequency is defined by the following formula:

*µ* = *α (p-q) + 2pqd*,

where *α* is the additive effect, *p* is the allele frequency of the synergetic allele, *q* is the allele frequency of the reduced allele, and *d* is the deviation caused by the dominant effect [20]. In the absence of allele interactions, it can be inferred that when the allele frequency (*p*) of a causal variation exceeds 0.50 (*d = 0*) or 0.30 (*d = α*), the population phenotype value (*µ*) will surpass the average level. Conversely, when the value of *p* is less than 0.50 or 0.30, the population phenotypic value will be lower than the mean. The present study focused on identifying the alleles associated with the smaller body size and slower growth rate observed in TIB populaiton. Assuming that the causal allele for this phenotype is P, with an allele frequency of *p* in the TIB population greater than 0.50 (*d* = 0) or greater than 0.30 (*d* = *α*), the mean value *µ* exceeded the average level. Consequently, the population phenotypic value for smaller body size and slower growth rate surpassed the mean level. In contrast, the population phenotypic value of smaller body size and slower growth rate in LW was smaller than the average level due to their larger body size and faster growth rate. This suggests that the allele frequency in LW would be less than 0.50 or 0.30. Furthermore, if each individual carries at least one causal allele for breed-specific traits, the probability *p* would be equal to or greater than 0.50. Taking these factors collectively, 0.50 could serve as the allele frequency cutoff value for screening SNPs associated with breed traits.

Thus, to investigate the potential causal relationship between SNPs and the lower growth rate of TIB, the allele frequencies of all SNPs in the TIB and LW populations were calculated separately using VCFtools(v4.2) [24], and SNPs with allele frequencies equal to or greater than 0.50 in the TIB population and simultaneously less than 0.50 in the LW population were initially extracted using a self-compiled Python program. Subsequently, we further identified TIB-specific SNPs by extracting SNPs with allele frequencies equal to zero in the LW population, and TIB-predominant SNPs by extracting SNPs with absolute allele frequency differences (△AF) between TIB and LW equal to or greater than 0.50 using a self-compiled Python program. The △AF values were determined by subtracting the allele frequency of each SNP in the TIB population from that in the LW population. All the TIB-specific and predominant SNPs were annotated to their corresponding genes using a Python program developed in-house. The density of TIB-specific SNPs was visualized using the R package CMplot. The percentage of TIB-specific SNPs on each chromosome, the distribution of TIB-specific SNPs based on allele frequency, the allele frequency distribution of TIB-predominant SNPs, and the location and functional classification of TIB-specific SNPs were visualized using OmicShare tools (www.omicshare.com/tools).

### Detecting LW-specific and predominant SNPs

Alternatively, if we consider a larger body size and faster growth rate as the desired phenotypes in LW, and assume that the causal allele for this phenotype is P, the allele frequency of *p* in LW would be greater than 0.50 (*d* = 0) or 0.30 (*d* = *α*). In contrast, the allele frequency in the TIB population would be less than 0.50 or 0.30. Therefore, to investigate the SNPs that contribute to faster growth in LW pigs, SNPs with allele frequencies equal to or greater than 0.50 in the LW population and simultaneously less than 0.50 in the TIB population were extracted using a self-compiled Python program. Subsequently, we further identified LW-specific SNPs by extracting SNPs with allele frequencies equal to zero in the TIB population, and LW-predominant SNPs by extracting SNPs with △AF between LW and TIB equal to or greater than 0.50 using a self-compiled Python program. The △AF values were determined by subtracting the allele frequency of each SNP in the LW population from that in the TIB population. All LW-specific and predominant SNPs were annotated to their corresponding genes using a Python program developed in-house. The density of LW-specific SNPs was visualized using the R package CMplot. The percentage of LW-specific SNPs on each chromosome, the distribution of LW-specific SNPs based on allele frequency, the allele frequency distribution of LW-predominant SNPs, and the location and functional classification of LW-specific SNPs were visualized using OmicShare tools (www.omicshare.com/tools).

### GO and KEGG pathway enrichment analysis of candidate genes

First, genes harboring TIB, LW-specific and predominant SNPs were defined as candidate genes. Due to the large number of candidate genes, GO (Gene Ontology) term and KEGG (Kyoto Encyclopedia of Genes and Genomes) pathway enrichment analysis were performed using the R package of gProfiler2 tools [25]with the parameters organism = “sscrofa”, ordered_query = FALSE, multi_query = FALSE, significant = TRUE, exclude_iea = FALSE, measure_underrepresentation = FALSE, evcodes = TRUE, user_threshold = 0.05, correction_method = “fdr”, domain_scope = “annotated”, custom_bg = NULL, numeric_ns = “”, sources = “GO: BP”, and as_short_link = FALSE. For the GO terms “cell component and molecular function” and “KEGG analysis”, the parameters used were as follows: sources = “GO: BP” modified as sources = “GO: CC”, sources = “GO: MF” and sources = “KEGG”. The genes enriched in GO terms and KEGG pathways related to skeletal muscle development were retained as potential genes involved in skeletal muscle development. The allele frequencies of SNPs in these potential genes were extracted from files of TIB- and LW-specific and predominant SNPs using a self-compiled Python program. Muscle related GO terms and the allele frequency of skeletal muscle development related genes in the TIB and LW populations were visualized using OmicShare tools (www.omicshare.com/tools). The haplotype blocks of skeletal muscle development-related genes were revealed using Haploview 4.2 [26].

### Selective sweep analysis

We calculated the genome-wide distributions of *F*_ST_ values and π-ratios (πLW/πTIB) between 44 TIB individuals and 29 LW individuals to assess the genomic regions with high genetic divergence and high differences in genetic diversity with a 50 kb sliding window size and a 10 kb step size using VCFtools (v4.2) [24]. The windows with the top 5% of *F*_ST_ values and π-ratios (πLW/πTIB) were considered to be candidate regions under strong selective sweeps and visualized using the R package ggPlot2. All candidate regions were then assigned to corresponding genes using in-house scripts. KEGG pathway and GO term analysis were performed using the online software g: Profiler [27]. The parameters of the g: Profiler tool (https://biit.cs.ut.ee/gprofiler/orth) were set as follows: organism set to “*sus scrofa*”, statistical domain scope set to “only annotated genes”, significance threshold set to “Benjamini-Hochberg FDR”, and user threshold set to “0.05”. Venn diagram was generated using the OmicStudio tools athttps://www.omicstudio.cn/tool.

## Results

### Genome sequencing and variations

In total, 73 whole-genome sequencing data were used in this study; These data consisted of 44 TIB pigs from Tibet, Sichuan, Gansu, and Yunnan Provinces, as well as 29 LW pigs. This dataset produced 16.47 billion raw reads, with a mean depth of 9.13× per individual and average genome coverage of 98.96% (Supplementary Table S1). Following variant calling and filtration, 21,594,848 and 15,210,134 SNPs were identified for TIB and LW, respectively. In total, 21,767,938 SNPs were obtained from the 73 individuals for subsequent analysis. These SNPs were categorized into 28 types, the large majority of which were intergenic region (51.62%) and intronic (43.70%) variants (Supplementary Table S2).

### TIB-specific and predominant SNPs

**Fig. 1:**
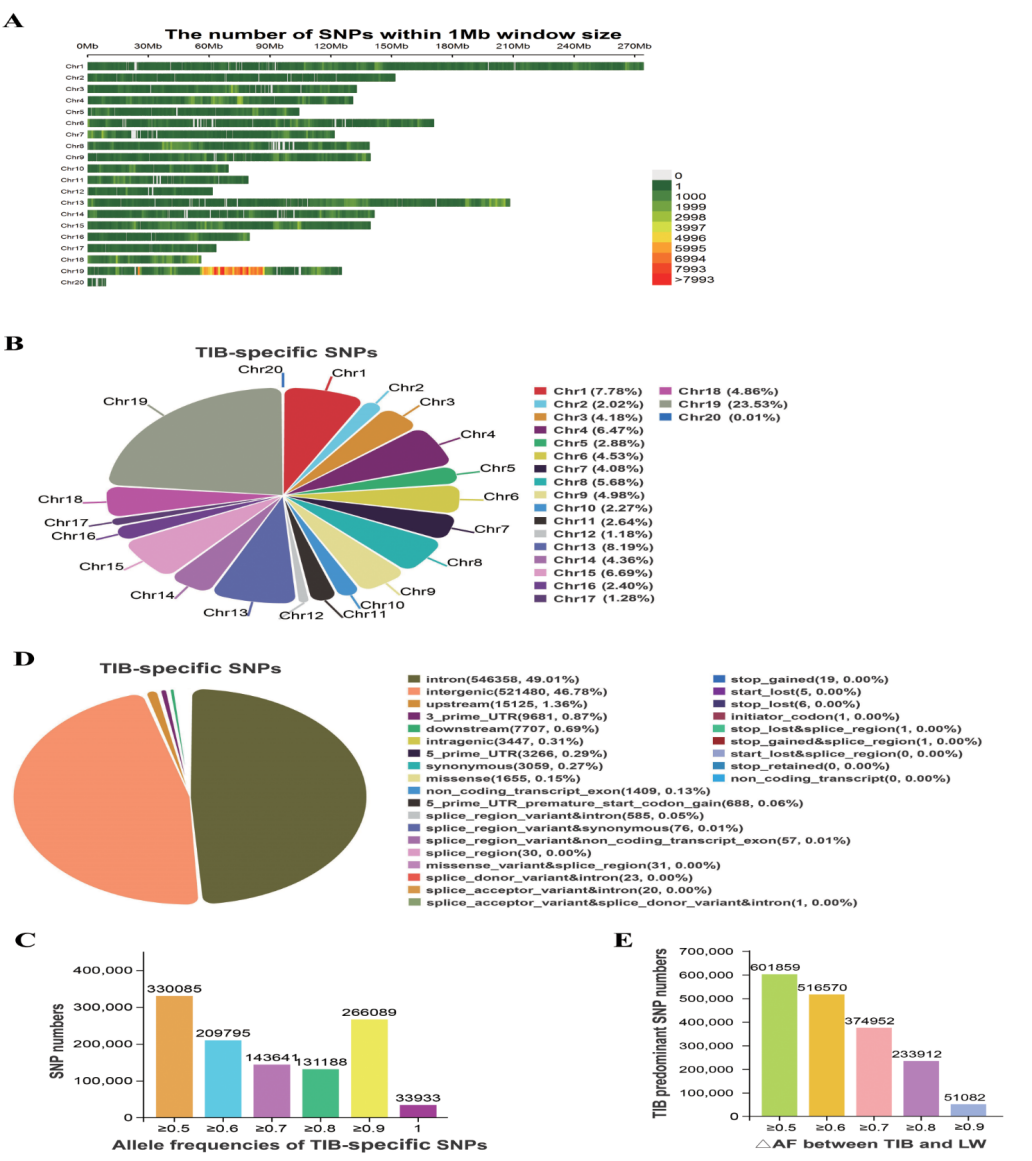
Summary of TIB-specific and predominant SNP metrics. **(A)** TIB specific SNP density for each chromosome; **(B)** Percentage of TIB-specific SNPs on each chromosome; **(C)** Allele frequency distribution of TIB-specific SNPs; **(D)** Location and functional classification of TIB-specific SNPs; **(E)** Distribution of TIB-predominant SNP △AF between TIB and LW

In this study, SNPs exhibiting allele frequencies equal to or greater than 0.50 in the TIB population, but with an allele frequency of zero in the LW population were designated as TIB-specific SNPs, and SNPs wih an allele frequency equal to or greater than 0.50 and simultaneously displaying a △AF between the TIB and LW populations equal to or exceeding 0.50 were classified as TIB-predominant SNPs. A total of 2,893,106 SNPs were detected, comprising 1,114,731 TIB-specific and 1,778,375 predominant SNPs. These TIB-specific SNPs accounted for 5.12% (1,114,731/21,767,938) of the total SNPs detected in this study and were found across all chromosomes (Fig. 1A). Intriguingly, the X chromosome (Chr19) exhibited the highest proportion of these SNPs (23.53%, 262,265/1,114,731) (Fig. 1B). The allele frequency of TIB-specific SNPs ranged from 0.50 to 1.0, with a greater proportion falling within the ranges of 0.50-0.60 (29.31%) and 0.90-1.0 (23.87%) (Fig. 1C). Furthermore, a total of 33,933 SNPs, accounting for 3.04% of the TIB-specific SNPs, exhibited an allele frequency of 1.0, indicating complete fixation in the TIB population. These specific SNPs were predominantly located in intergenic (46.78%) and intronic (49.01%) regions, with 3,059 synonymous and 1,655 missense variations observed (Fig. 1D). All the TIB-specific SNPs could be annotated to 18,697 genes. A total of 1,778,375 TIB-predominant SNPs were identified, which accounted for 8.17% (1,778,375/21,767,938) of the total SNPs identified in this study. The △AF of these TIB-predominant SNPs ranged from 0.50 to 1.0, with the majority falling within the range of 0.50-0.60 (Fig. 1E). Furthermore, these TIB-predominant SNPs could be annotated to 22,482 genes.

### LW-specific and predominant SNPs

**Fig. 2:**
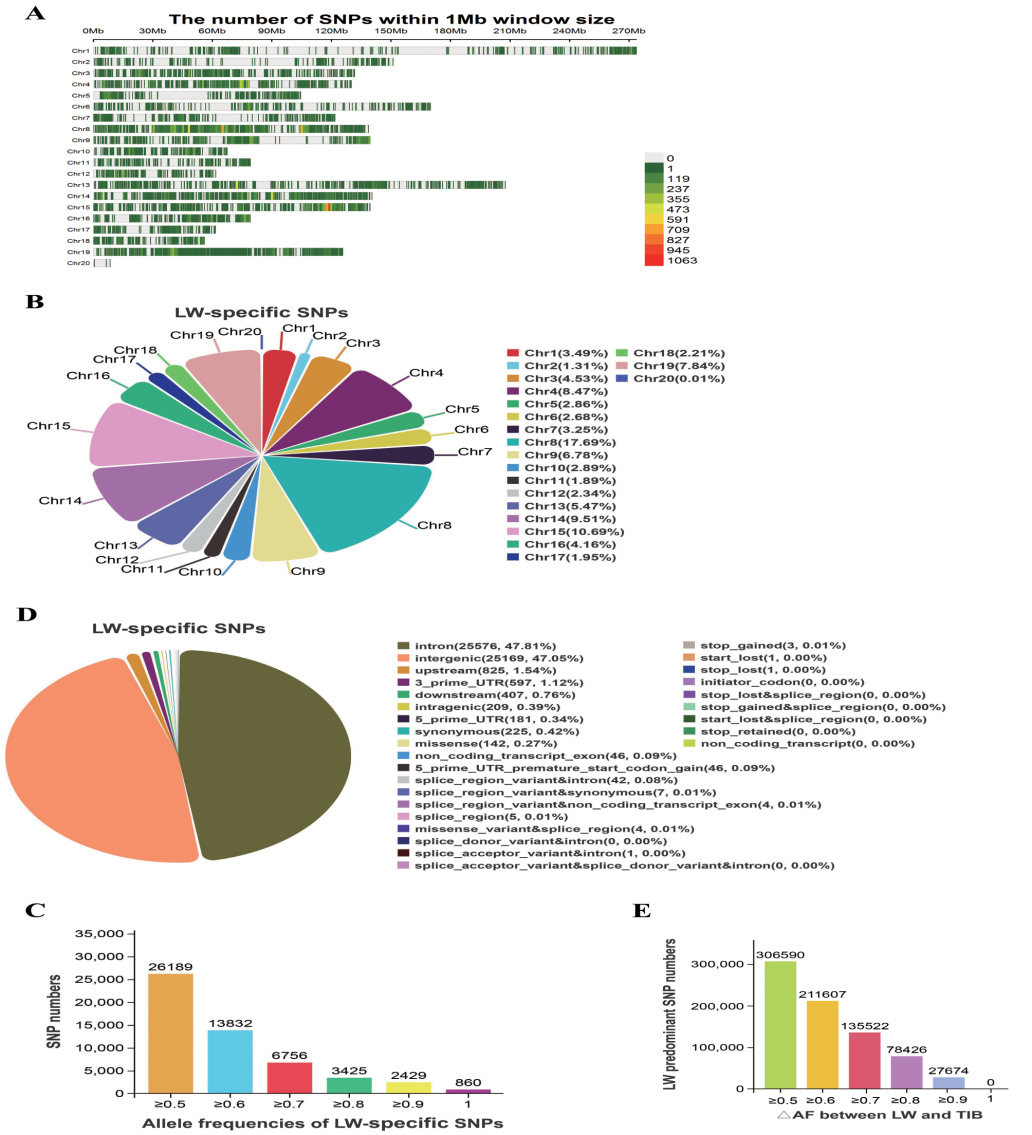
Summary of LW-specific and predominant SNP metrics. **(A)** LW-specific SNP density on each chromosome; **(B)** Percentage of LW-specific SNPs on each chromosome; **(C)** Allele frequency distribution of LW-specific SNPs; **(D)** Location and functional classification of LW-specific SNPs; **(E)** Distribution of LW-predominant SNP △AF between LW and TIB

In addition to investigate TIB-specific and predominant SNPs, we also examined LW-specific and predominant SNPs. SNPs exhibiting allele frequencies equal to or greater than 0.50 in the LW population, while having an allele frequency of zero in the TIB population, were designated as LW-specific SNPs, and SNPs having an allele frequency equal to or greater than 0.50, and simultaneously displaying a △AF between the LW and TIB populations equal to or exceeding 0.50 were classified as LW-predominant SNPs. A total of 813,310 SNPs were detected in the LW population, containing 53,491 LW-specific SNPs and 759,819 predominant SNPs. The LW-specific SNPs accounted for 0.25% (53,491/21,767,938) of the total SNPs in this study and were found on all chromosomes (Fig. 2A), with chromosome 8 having the highest proportion (9,465 SNPs, 17.69%) (Fig. 2B). The allele frequencies of these LW-specific SNPs ranged from 0.50 to 1.0, with a greater proportion falling within the ranges of 0.50-0.60 (48.96%) (Fig. 2C). Furthermore, 1.61% of the LW-specific SNPs (860) were found to have an allele frequency of 1.0 and completely fixed in the LW population. These specific SNPs were predominantly intronic (47.81%, *n* = 25,576) and intergenic region variations(47.05%, *n* = 25,169), with a smaller proportion of synonymous (225) and missense (142) variations (Fig. 2D). All LW-specific SNPs could be annotated to 6,834 genes. A total of 759,819 LW-predominant SNPs were detected, representing 3.49% (759,819/21,767,938) of the total SNPs in this study. The △AF of the LW-predominant SNPs ranged from 0.50 to 1.0, with most of the numbers falling within the range of 0.50-0.60 (Fig. 2E). Furthermore, these LW-predominant SNPs could be annotated to 16,444 genes.

### GO enrichment analysis of candidate genes to identify skeletal muscle development-related genes

We aggregated the genes containing TIB, LW-specific and predominant SNPs and designated them candidate genes. A total of 24,560 candidate genes were obtained. GO enrichment analysis of the candidate genes revealed that 2,925 biological process, 313 molecular function and 324 cell component terms were overrepresented. The overrepresented biological process terms were related to reproduction, immune system, neuron system development, lung development, heart system, pigmentation, lipid metabolic, and responsive to UV radiation, as detailed in Supplementary Table S3; the molecular function terms included ion binding, ATP binding, lipid binding, protein domain-specific binding, actin binding, ubiquitin-like protein ligase binding, and insulin-like growth factor binding (Supplementary Table S4); the cell component terms were related to the nucleoplasm, endomembrane system, cell projection, cytoskeleton, extracellular matrix, actin cytoskeleton, and several others (Supplementary Table S5).

**Fig. 3:**
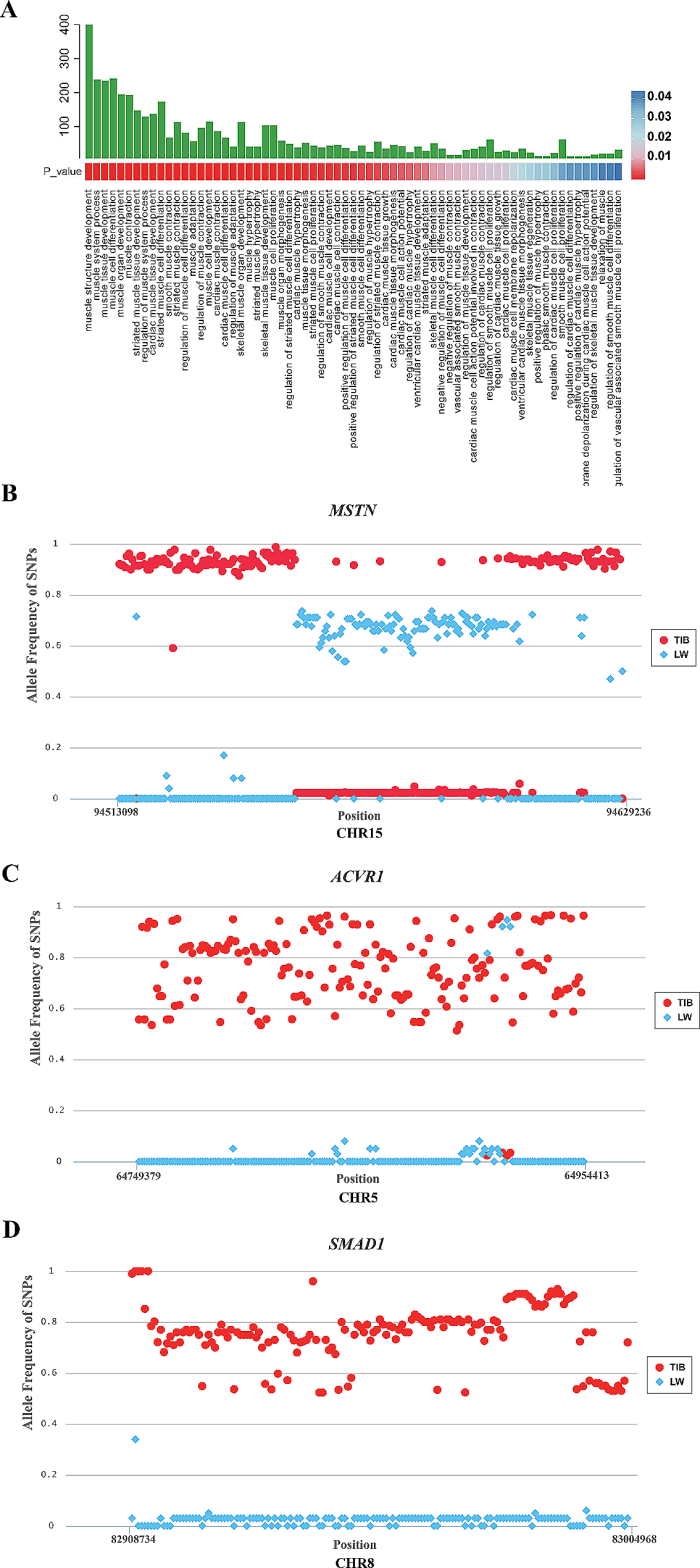
Muscle-related GO terms and genes identified by GO analysis. Muscle development related GO terms for candidate genes **(A)**, and SNP allele frequency of *MSTN* **(B)**, *ACVR1*** (C)** and *SMAD1*** (D)** genes in TIB and LW populations

The analysis revealed that the GO terms associated with biological processes pertaining to skeletal muscle development provided more comprehensive information. Specifically, a total of 67 biological process terms related to various types of muscle tissue, such as striated, skeletal, smooth, and cardiac tissue, were identified (Fig. 3A). Given that skeletal muscle falls under the category of striated muscle, our attention was directed toward the biological process GO terms specifically related to skeletal and striated muscle. Notably, eight out of the 67 terms were found to be involved in the biological processes of skeletal and striated muscle development (striated muscle cell differentiation, striated muscle hypertrophy, regulation of striated muscle cell differentiation, striated muscle cell proliferation, positive regulation of striated muscle cell differentiation, skeletal muscle cell differentiation, striated muscle tissue development, and skeletal muscle tissue development). In addition, myoblast differentiation and fusion were also significantly overrepresented in the biological process related GO terms. A total of 291 genes were identified to be involved in the 8 biological processes related to skeletal and striated muscle, as well as myoblast differentiation and fusion related biological processes.

**Fig. 4:**
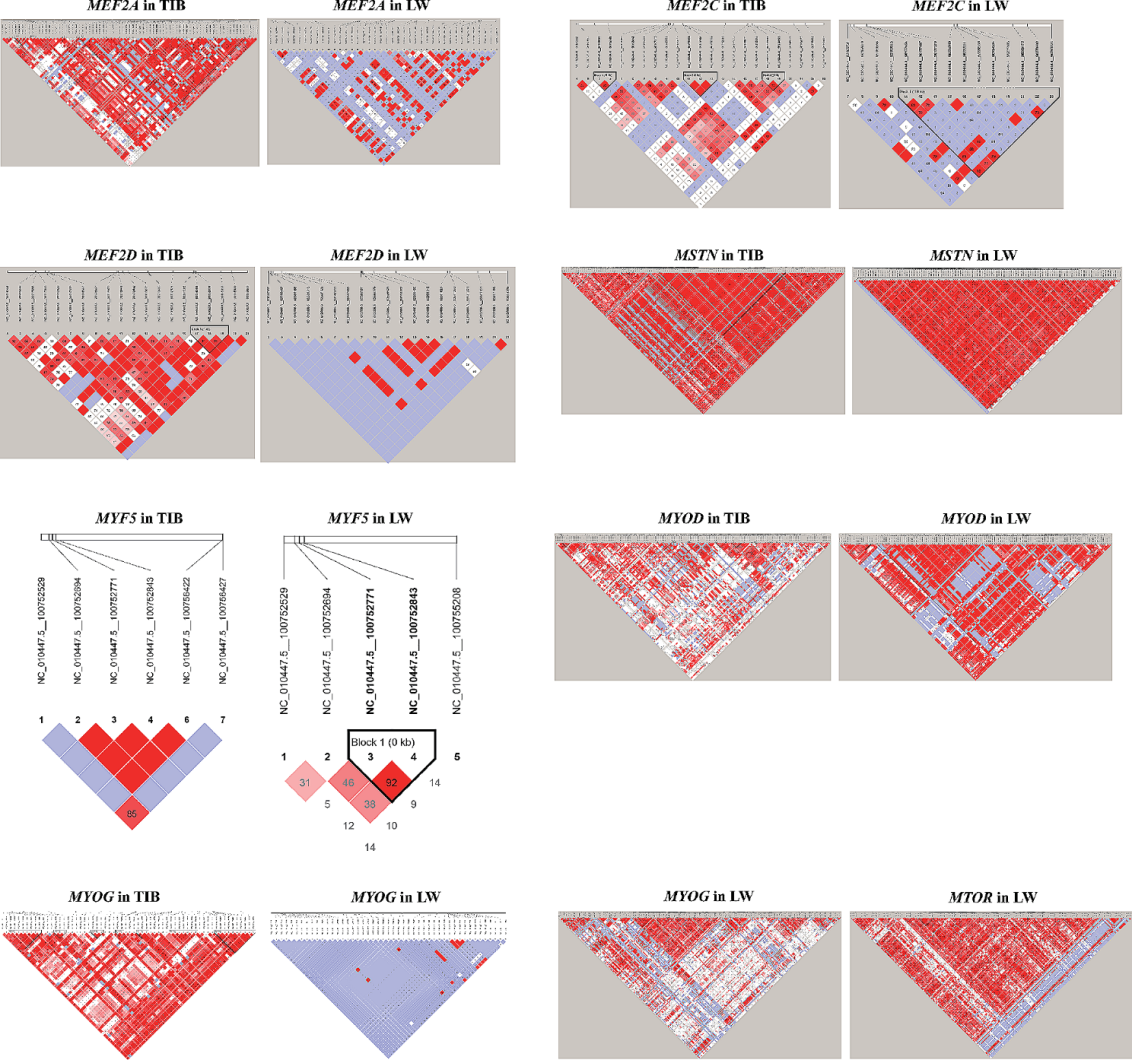
Haplotype blocks of * MEF2A*, *MEF2C*, *MEF2D*, *MSTN*, *MYF5*, *MYOD*, *MYOG*, *MTOR* genes in TIB and LW populations

These 291 genes contained a total of 82,349 SNPs, including 17,286 TIB-specific and 40,864 predominant SNPs and 1,497 LW-specific and 22,702 predominant SNPs (Supplementary Table S6). Moreover, 53 missense SNPs were detected in 32 genes, including *FOS*, *MYOM2*, *MYOCD*, *MYOC*, and *TANC1*, and 249 synonymous SNPs were observed in 97 genes, such as *MYOM2*, *MYOF*, *YAP1*, *MYH9*, *MTOR*, *MYH7*, *MYOCD*, *TANC1*, *MYOC*, and *MYF5*. Relatedly, 1566 SNPs were found within the regulatory regions (including upstream region, downstream region, 5’ UTR, and 3’ UTR) of 211 genes, such as *MSTN*, *MYOZ1*, *MYF6*, *MYOD1*, *SMAD1*, *MYF5*, *SMAD4*, *MYH9*, *MYOC*, *MTOR*, *MYOG*, *MYOM2*, and *MEF2D*. Furthermore, there were 259 TIB-specific SNPs with allele frequencies equal to or greater than 0.95; these SNPs could be annotated to 15 genes (*MSTN*, *SMAD1*, *ERBB4*, *DOCK2*, *PLEKHM3*, *TANC1*, *ACVR1*, *PDGFRA*, *SGCB*, *ZFPM2*, *EPAS1*, *SMYD3*, *CACNA1S*, *NEBL*, and *PAXBP1*). In addition, 5 LW-specific SNPs with allele frequencies equal to or greater than 0.95 were identified in the intergenic regions of *MTPN* and *TCF7L2*. Among these genes, several were found to play crucial roles in skeletal muscle development. These included all four members of the MRF family (*MYOD*, *MYF5*, *MYOG*, and *MYF6*), as well as three members of the MEF2 family (*MEF2A*, *MEF2C*, and *MEF2D*). Additionally, genes involved in muscle growth inhibition, namely *MSTN*, *SMAD1*, and *ACVR1*, as well as the gene responsible for protein synthesis, *MTOR*, were implicated. Notably, *MSTN, SMAD1, and ACVR1* exhibited significant differences in allele frequency between our TIB and LW populations (Fig. 3B, C, D). Furthermore, *MYF5* exhibited a TIB-specific synonymous SNP with an allele frequency of 0.84, along with six upstream region SNPs; Similarly, the *MYOD*, *MYOG*, and *MYF6* genes also exhibited several upstream region SNPs. Further investigation of haplotype blocks revealed that *MYOD*, *MYF5*, *MYOG*, *MEF2A*, *MEF2C*, *MEF2D*, *MSTN*, and *MTOR* exhibited different haplotype blocks between the TIB and LW populations (Fig. 4).

### KEGG pathway analysis of candidate genes to identify skeletal muscle development-related genes

**Fig. 5:**
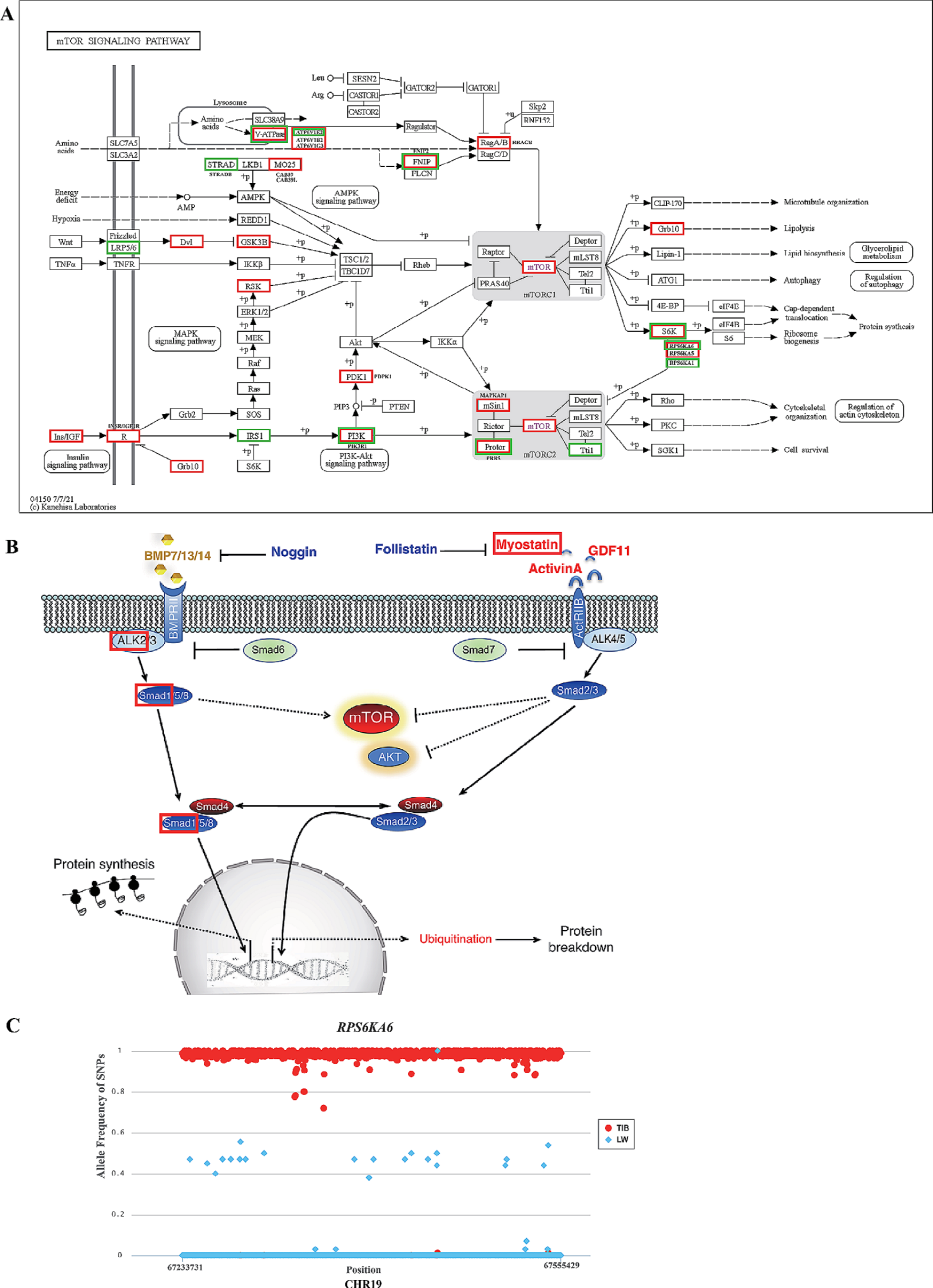
Genes involved in the mTOR and TGFβ/myostatin/activin/BMP signaling pathways. **(A)** Ten genes involved in the mTOR signaling pathway harbored missense variations (green box) and 19 genes harbored TIB-specific SNPs with allele frequency > = 0.95 (red box); **(B)** Genes involved in TGFβ/myostatin/activin/BMP signaling pathway harbored TIB-specific SNPs with allele frequency > = 0.95(red box); **(C)** SNP allele frequency of *RPS6KA6* genes in TIB and LW populations. The mTOR signaling pathway map were downloaded from https://www.kegg.jp/kegg/pathway.html [34], and the TGFβ/myostatin/activin/BMP signaling diagram was adapted from [35]

The KEGG pathway analysis revealed that a total of 36 KEGG pathways were over-represented, including the PI3K-Akt signaling pathway, protein digestion and absorption, the TGF-beta signaling pathway, the calcium signaling pathway, the Wnt signaling pathway, the mTOR signaling pathway, and others (Supplementary Table S7). Several signaling pathways, including the PI3K-Akt signaling pathway [28, 29], the TGF-beta signaling pathway [30], the calcium signaling pathway [31, 32], the Wnt signaling pathway [33] and the mTOR signaling pathway, are involved in skeletal muscle development. Among these pathways, the mTOR and TGFβ/myostatin/activin/BMP signaling pathways play significant roles in regulating protein synthesis, with mTOR acting as a positive regulator and TGFβ/myostatin/activin/BMP acting as a negative regulator [11]. Therefore, our study focused on the genes involved in the mTOR signaling pathway [34] (Fig. 5A) and the TGFβ/myostatin/activin/BMP signaling pathway [35] (Fig. 5B), which are partial segments of the TGF-beta signaling pathway.

A total of 106 genes were implicated in the mTOR signaling pathway, encompassing 23,925 SNPs. Among these SNPs, 9,319 TIB-specific and 10,040 predominant SNPs, as well as 556 LW-specific and 4010 predominant SNPs, were present (Supplementary Table S8). Moreover, 15 missense variations were found in 10 genes, including *RPS6KA6*, *RPS6KA1*, *STRADB*, *ATP6V1E2*, *PRR5*, *FNIP2*, *TTI1*, *IRS1*, *LRP5*, and *PIK3R1;* their locations in the mTOR signaling pathway are depicted in Fig. 5A with a green box. Notably, three TIB-specific SNPs were observed in the *RPS6KA6* gene, with an average allele frequency of 0.99. Ninety synonymous SNPs were observed in 45 genes, namely *PRS6KA6*, *IGF1*, *IGF1R*, *IRS1*, *MTOR*, and *GRB10*. Relatedly, 828 variations in regulatory regions were observed in 84 genes, including *IGF1*, *RPS6KA6*, *GRB10*, *MTOR*, *IRS1*, and *GRB2.* Furthermore, 24 SNPs were found in the splice-regions of 17 genes, specifically *RPS6KA6*, *MTOR*, *INSR*, and *IGF1R*. Importantly, 3,804 TIB-specific SNPs with allele frequencies equal to or greater than 0.95 were detected locating in 19 genes, including *FNIP2*, *GRB10*, *RPS6KA6*, *RPS6KA3*, *RRAGB*, *GSK3B*, *IGF1*, *IGF1R*, *MAPKAP1*, *PIK3R1*, *PRR5*, *DVL3*, *CAB39L*, *CAB39*, *ATP6V1E2*, *ATP6V1B2*, *ATP6V1G3*, and *PDPK1*, the positions of which are in the mTOR signaling pathway, as depicted in Fig. 5A with a red box. Additionally, 4 LW-specific SNPs with allele frequencies equal to or greater than 0.95 were identified and annotated to the *MAPKAP1* and *FNIP2* genes. We further observed 1,858 TIB-specific SNPs within a 182 kb region of the *RPS6KA6* gene, with an average allele frequency of 0.98, indicating significant disparity in allele frequencies between the TIB and LW populations (Fig. 5C). The 1,858 TIB-specific SNPs included upstream, 3’ UTR, 5’ UTR, missense, synonymous, and intron varirants.

The TGFβ/myostatin/activin/BMP signaling pathway was referenced from study by Sartori et al. [35]. The pathway includes 22 genes of *BMP7*, *Follistatin* (*FST*), *Myostatin (MSTN)*, *ActivinA* (*INHBA*), *ALK4* (*AVCR1B*), *ActRIIB* (*AVCR2B*), *BMPR2*, *GDF11*, *ALK5* (*TGFBR1*), *ALK2* (*ACVR1*), *ALK3* (*BMPR1A*), *SMAD1*, *SMAD2*, *SMAD3*, *SMAD4*, *SMAD5*, *SMAD6*, *SMAD7*, *SMAD9* (*SMAD8*), *Noggin* (*NOG*), *BMP13*, and *BMP14*. Except for *BMP13* and *BMP14*, the other 20 genes were found to be included in the candidate genes (Fig. 5B). These 20 genes contained 4,850 SNPs, which included intergenic (2,785), intron (1,874), synonymous (7), splice-region (1) and regulatory region varirants (183). Notably, the genes *MSTN*, *SMAD1* and *ACVR1* exhibited TIB-specific SNPs with allele frequencies equal to or greater than 0.95; their positions in the pathway was shown in Fig. 5B with a red box.

### Skeletal muscle development-related genes identified by selective sweep analysis

**Fig. 6:**
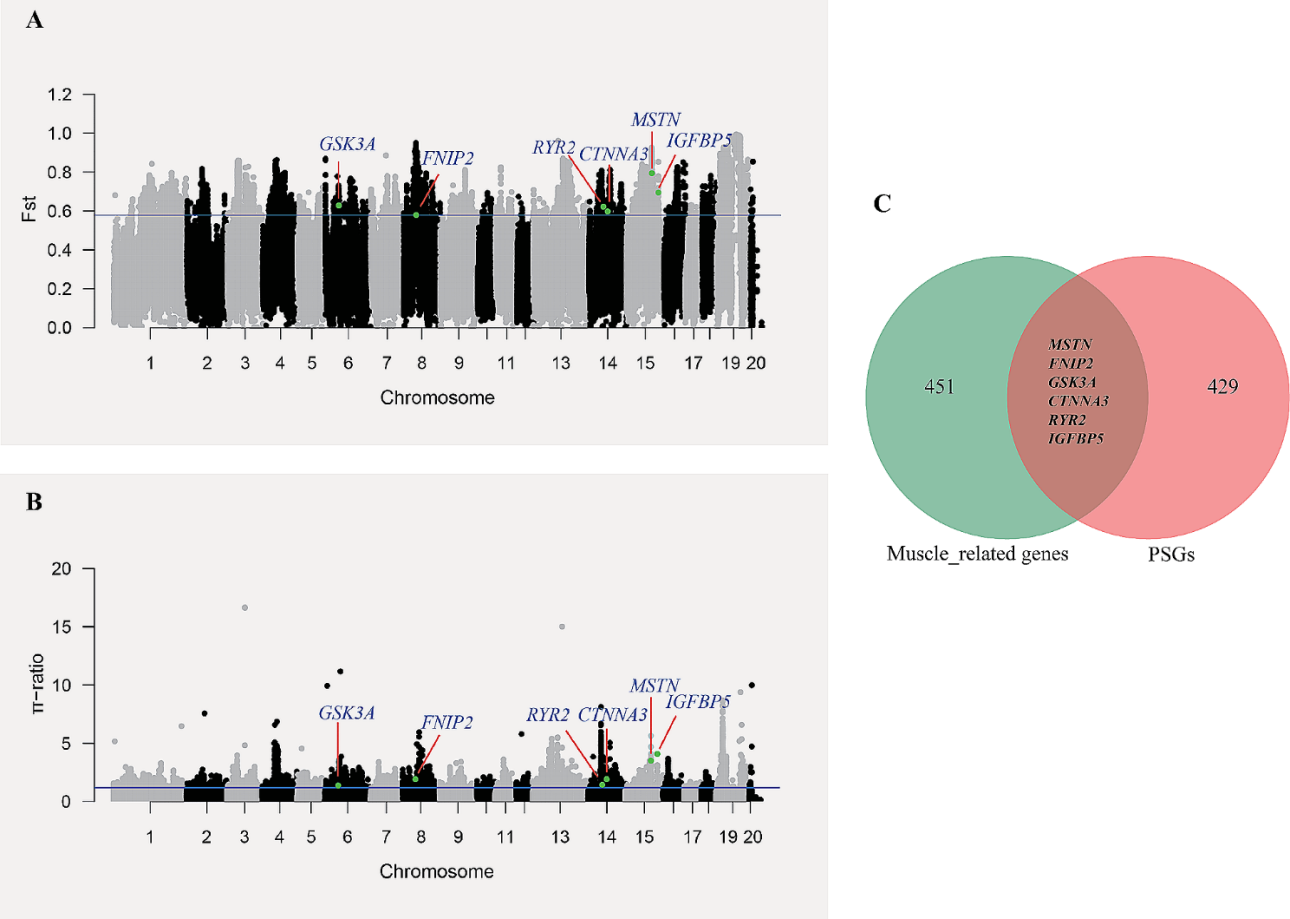
Identifying skeletal muscle development related genes by selective sweep analysis. **(A)** Distribution of *F*_ST_ values calculated in 50 kb sliding window size with 10 kb step size between TIB and LW; **(B)** π-ratio (π_LW_/π_TIB_) was calculated by π value in LW / π value in TIB in 50 kb window size with 10 kb step size; **(C)** Overlap genes between PSGs and muscle related genes enriched in 67 muscle related biological process by GO analysis for candidate genes was showed by Venn diagram. Each dot represented 50 kb sliding window size and 10 kb step size, within which the average *F*_ST_ value and π-ratio (π_LW_/π_TIB_) were calculated. The blue line was the top 5% of *F*_ST_ (0.58) and π-ratio (π_LW_/π_TIB_) (1.20) line. Genes visualized in **(A)** and **(B)** were the overlap genes

To examine genes associated with skeletal muscle development identified through the selective sweep analysis method, we conducted a search of the genome for regions exhibiting high allele frequency differentiation and nucleotide diversity. This was achieved by calculating the *F*_ST_ and π-ratio values between our TIB and LW populations using a window size of 50 kb and a step size of 10 kb. A total of 435 genes, representing the top 5% of regions in terms of both the *F*_ST_ value and π-ratios (π_LW_/π_TIB_), were identified as putatively selected genes (PSGs) (Supplementary Table S9) (Fig. 6A and B). All the PSGs were subjected to KEGG and GO enrichment analysis, leading to the identification of a total of 10 overrepresented GO terms of biological processes; however, no terms showed direct relation to skeletal muscle development. Therefore, to ascertain the links of PSGs to skeletal muscle development, we conducted an overlap analysis to identify the genes common to both PSGs and genes implicated in muscle development-related biological processes GO terms (67 terms), as well as in the mTOR and TGFβ/myostatin/activin/BMP signaling pathways. The results of this analysis revealed an overlap of six genes (*MSTN*, *FNIP2*, *GSK3A*, *CTNNA3*, *RYR2*, and *IGFBP5*) as depicted in Fig. 6C).

## Discussion

In this study, we detected specific and predominant SNPs in the TIB and LW populations by screening allele frequency for each SNP at the whole-genome level to identify genes potentially related to skeletal muscle development. Our analysis revealed a total of 2,893,106 (13.29%) specific and predominant SNPs were detected in the TIB population and 813,310 (3.74%) in the LW population. These SNPs accounted for 17.03% of all SNPs identified in this study. Specifically, 1,114,731 (5.12%) TIB-specific SNPs and 53,491 (0.25%) LW-specific SNPs were detected, which may contribute to the unique traits of each breed and the divergent phenotypes observed between the TIB and LW populations. All the TIB- and LW-specific and predominant SNPs could be annotated to 24,560 genes in total, far more than the 435 genes identified through selective sweep analysis using empirical distributions of cutoff points at the 95th percentile, a commonly used empirical cutoff value. The set of 24,560 candidate genes exhibited significant enrichment in 2,925 biological processes-related GO terms, encompassing not only skeletal muscle development but also various other biological processes, such as pigmentation, immunity, reproductive capacity, and fat deposition. This result aligns with the observed phenotypic disparities between TIB and LW pigs, which extend beyond growth and body size to encompass coat color, disease resistance, fertility, and subcutaneous and intramuscular fat deposition.

### Skeletal muscle growth-related genes identified by GO enrichment analysis

The process of skeletal muscle development implicates an increase in both the number and size of muscle cells; this process is known as muscle fiber hyperplasia and hypertrophy. Previous research has demonstrated that, compared with miniature pigs, LW pigs contain a 173% greater quantity of muscle fibers in the *M. semitendinosus* [36]. Additionally, fast-growing lean pigs have more myofibers and larger myofibers than slow-growing fat pigs [5]. Therefore, our investigation focused on genes implicated in the biological mechanisms of myoblast differentiation, and fusion, as well as skeletal and striated muscle differentiation, proliferation, and hypertrophy. The MRFs (*MyoD*, *Myogenin*, *Myf5*, *Myf6*) and MEF2 (*MEF2A*, *MEF2B*, *MEF2C*, *MEF2D*) protein families have been identified to play significant roles in these processes [6–9]. Specifically, *MyoD* and *Myf5* are involved in the specification of embryonic mesodermal progenitor cells, facilitating their differentiation into myoblasts. On the other hand, *MyoG* and either *MyoD* or *Myf6 *are needed for the differentiation of myoblasts into myocytes. Furthermore, the collaboration between *MyoD* and members of the MEF2 family is essential for the transcription activation of the majority of skeletal muscle genes [6, 7, 9]. The findings of our study indicate that all four members of the MRFs and three members of the MEF2 family (*MEF2A*, *MEF2C*, and *MEF2D*) exhibited marked differences in allele frequency and haplotype blocks between TIB and LW. The protein *MSTN* is known to play a crucial role in the inhibitory regulation of postnatal muscle fiber hypertrophy through its interaction with its receptor and subsequent activation of signaling pathways that impede protein synthesis [11, 35]. For example, transgenic Meishan pigs exhibit increased skeletal muscle mass associated with a loss-of-function mutation in the *MSTN* gene [37]. Two upstream region SNPs and different haplotype blocks were found between the TIB and LW populations in the *MSTN* gene and its upstream intergenic region in this study. Furthermore, the mTOR gene, which is responsible for encoding the kinase mTOR, a pivotal gene involved in protein synthesis and degradation, was found to harbor seven synonymous, five upstream, three splice region intronic and 225 intronic SNPs. The kinase mTOR plays a crucial role in two distinct complexes, namely mTORC1, which governs protein synthesis, cell growth and proliferation, and mTORC2, which acts as a regulator of the actin cytoskeleton, promoting cell survival and cell cycle progression. Previous studies have reported that the conditional deletion of mTOR, accompanied by the expression of catalytically inactive mTOR, leads to a decrease in growth rate beginning 1 week after birth [35].

Furthermore, we observed many other genes covering multiple missense, synonymous, and regulatory region SNPs. These genes included *SGCB*, *MYOM2*, *MYOCD*, *MYOC*, *MYOF*, *YAP1*, *MYH9*, *MYH7*, *SMAD1*, and *SMAD4*, which have been reported to be related to skeletal muscle development. *SGCB* is responsible for encoding the beta subunit of the sarcoglycan protein complex, a collection of transmembrane proteins that play a crucial role in stabilizing muscle fiber membranes and connecting the muscle cytoskeleton to the extracellular matrix; deficiencies or mutations in *SGCB* can result in a specific form of muscular dystrophy called limb girdle muscular dystrophy type 2E, which is characterized by progressive muscle weakness and degeneration [38]. *MYOM2*, a member of the MYOM gene family, is predominantly expressed in fast-twitch muscle fibers; the MYOM protein family functions as an integral constituent of the M-band, facilitating the crosslinking of myosin filaments to confer stability to the sarcomere [39]. *MYOCD* is transiently expressed in skeletal muscle progenitor cells located in somites, and a significant portion of skeletal muscle originates from cell lineages that express Myocd; nevertheless, instead of inducing the expression of genes specific to skeletal muscle, Myocd acts as a transcriptional repressor of Myog, thereby impeding the process of skeletal muscle differentiation [40]. *YAP1* is a downstream nuclear effector of the Hippo signaling pathway and is implicated in various physiological processes, such as development, growth, repair, and homeostasis; additionally, *YAP1* is involved in the promotion of muscle hypertrophy through its overexpression, which results in increased protein synthesis; conversely, depletion of *YAP1* can induce muscle atrophy by diminishing protein synthesis [12].

Breed-specific SNPs with nearly fixed or fixed allele frequencies have been found to be associated with breed-specific traits [41]. A total of 259 TIB-specific SNPs were identified in the TIB population, with allele frequencies equal to or greater than 0.95. These SNPs were annotated to genes related to skeletal muscle development, including *MSTN*, *SMAD1*, *ERBB4*, *DOCK2*, *PLEKHM3*, *TANC1*, *ACVR1*, *PDGFRA*, *SGCB*, *ZFPM2*, *EPAS1*, *SMYD3*, *CACNA1S*, *NEBL*, and *PAXBP1*. Therefore, these genes may play significant roles in the smaller body size and slower growth rates observed in TIB pigs compared to LW pigs.

### Skeletal muscle growth-related genes identified by KEGG pathway analysis

Muscle hypertrophy results from protein synthesis exceeding protein degradation [37]. The mammalian target of rapamycin (mTOR) and the TGFβ/myostatin/activin/BMP are two major signaling pathways that control protein synthesis and act as a positive and negative regulator of muscle hypertrophy [11]. The map of mTOR signaling pathway shows that the insulin/IGF-PI3K-Akt-mTORC1, insulin/IGF-RAS-MAPK-ERK-mTORC1, amino acids-mTORC1, AMPK-mTORC1, Wnt-mTORC1 and insulin/IGF-PI3K-mTORC2 subpathways are included in this signaling pathway. This study detected 106 genes harboring 9,319 TIB-specific and 10,040 predominant SNPs, and 556 LW-specific and 4,010 predominant SNPs involved in the mTOR signaling pathway. Muscle protein synthesis in piglets is highly sensitive to alterations in insulin and amino acid levels that occur after feeding, and the initiation of translation is facilitated by the activation of mTORC1 by insulin and amino acids [37]. In this study, we observed that genes harboring missense and specific SNPs with allele frequencies equal to or greater than 0.95 were associated mainly with pathways involving insulin and amino acids (insulin/IGF-PI3K-Akt-mTORC1 and amino acids-mTORC1).

The interaction of insulin and/or insulin-like growth factor-1 (IGF1) with their respective receptors, INSR and IGF1R, which locate on the cell membrane, initiates the phosphorylation of insulin receptor substrate (IRS1). IRS1 serves as an adapter protein that activates phosphatidylinositol 3-kinase (PI3K), which in turn recruits 3-phosphoinositide-dependent protein kinase 1 (PDK1) and phosphorylates protein kinase B or Akt. Downstream of Akt, the tuberous sclerosis complex (TSC1-TSC2) acts as a target and inhibits the small G protein Ras homolog enriched in the brain (RHEB), which serves as a regulator of mTOR [37, 42]. *IGF1*, *IGF1R*, *INSR*, *IRS1*, *PI3K* (*PIK3R1*), and *PKD1* (*PDPK1*) were found to harbor missense or specific SNPs with allele frequencies equal to or greater than 0.95, with *PI3K* harboring both types of SNPs.

Amino acids accumulate within the lysosomal lumen and serve as a signal to the vacuolar V-ATPase through an ‘inside-out’ mechanism. This V-ATPase is responsible for controlling the binding of the RAG GTPase-Ragulator [43]. Additionally, GTPases are recruited to lysosomes by Ragulator. Simultaneously, amino acids enhance Ragulator’s GEF activity toward GDP-bound Rag A/B as well as FLCN-FNIP1/2 and LRS’s GAP activity towards GTP-bound Rag C/D. Active Rag GTPases can then recruit mTORC1 to the lysosome, where it interacts with GTP-bound RHEB, initiating mTORC1 signaling [44]. Three V-ATPase subunits (*ATP6V1E2*, *ATP6V1B2*, and *ATP6V1G3*), *FNIP2*, and *RRAGB* were observed to have missense or specific SNPs with allele frequencies equal to or greater than 0.95, and *ATP6V1E2* was found to have both of these types of SNPs.

The mTORC1 complex interacts with its two primary substrates, eukaryotic initiation factor (eIF) 4E binding protein 1 (4E-BP1) and p70 ribosomal protein S6 kinase 1 (S6K1), to initiate the process of protein synthesis. Notably, the phosphorylation of 4E-BP1 and S6K1 plays a significant role in regulating the rate-limiting step of protein synthesis [37]. Our data revealed the presence of missense or specific SNPs with allele frequencies greater than or equal to 0.95 in three S6K genes (*RPS6KA6*, *RPS6KA5* and *RPS6KA1*). Specifically, we observed 1858 TIB-specific SNPs with an allele frequency of 0.98 in a 182 kb region encompassing the *RPS6KA6* gene. These specific SNPs were observed in our dataset and exhibited various functional types, including upstream, 3’ UTR, 5’ UTR, missense, synonymous, intron, and synonymous variants. This observation suggested that *RPS6KA6* may contribute to the disparity in muscle growth between the TIB and LW populations.

## Conclusion

In this study, 2,893,106 (13.29%) specific and predominant SNPs in the TIB population, and 813,310 (3.74%) in the LW population were detected and annotated to 24,560 genes. A total of 291 genes were found to be involved in the biological processes related to skeletal muscle differentiation, proliferation, hypertrophy, and myoblast differentiation and fusion; 106 genes were involved in the mTOR signaling pathway, a critical positive signaling pathway for muscle growth, and 20 genes were included in the TGFβ/myostatin/activin/BMP signaling pathway, a negative signaling pathway associated with muscle fiber hypertrophy. Among these genes, several have been extensively studied and are considered crucial for skeletal muscle development; they included MRF and MEF2 family members; the muscle growth inhibitors *MSTN*, *ACVR1*, and *SMAD1;* and the protein synthesis genes *IGF1, IGF1R*, and *mTOR*. Additionally, numerous other genes, such as *RPS6KA6, MYOM2, MYOCD, YAP1, SGCB, ATP6V1E2, ATP6V1B2*, and *ATP6V1G3*, contained missense, synonymous or specific SNPs fixed or nearly fixed. These genes may also play significant roles in the differences observed in skeletal muscle growth between TIB and LW populations. This study employed an effective methodology to rigorously identify the potential genes associated with skeletal muscle development, and the findings offer valuable insights into the genetic underpinnings of skeletal muscle development and hold considerable implications for enhancing commercial meat production through pig breeding.

### Electronic supplementary material

Below is the link to the electronic supplementary material.

Supplementary Material 1

Supplementary Material 2

Supplementary Material 3

Supplementary Material 4

Supplementary Material 5

Supplementary Material 6

Supplementary Material 7

Supplementary Material 8

Supplementary Material 9

Supplementary Material 10

## Data Availability

The raw data was deposited in the NCBI database using SRA accession numbers SRR17839521 - SRR18086562.

## Abbreviations

TIB: Tibetan pigs
LW: Large white pigs
△AF: Absolute allele frequency difference
GO: Gene Ontology
KEGG: Kyoto Encyclopedia of Genes and Genomes
MRF: Myogenic regulatory factor
MEF2: Myocyte enhancer factor 2
SNP: Single nucleotide polymorphism
