# Supplementary material for: Whole-genome SNP allele frequency differences between Tibetan and Large white pigs reveal genes associated with skeletal muscle growth
Source: BMC Genomics. 2024 Jun 12;25:588. doi: 10.1186/s12864-024-10508-7 (PMC11167949; doi:10.1186/s12864-024-10508-7)
Supplement: Supplementary file 8 — Supplementary Material 8 [file 12864_2024_10508_MOESM8_ESM.docx]

## Table S1. The sample and sequence information.

## Table S2. Information of SNP categories by type.

## Table S3. Enriched GO terms of biological process for candidate genes.

## Table S4. Enriched GO terms of molecular functions for candidate genes.

## Table S5. Enriched GO terms of cell components for candidate genes.

## Table S6. SNPs and genes involved in biological processes of skeletal and/ or striated muscle development and myoblast differentiation and fusion.

## Table S7. Enriched KEGG pathways for candidate genes.

## Table S8. SNPs and genes identified in mTOR KEGG pathways.

## Table S9. List of genes in the selected overlapping regions of top 5% *F*_ST_ and Pai ratio (LWTIB).
